# Correction to ‘Genome instability and pressure on non-homologous end joining drives chemotherapy resistance via a DNA repair crisis switch in triple negative breast cancer’

**DOI:** 10.1093/narcan/zcab041

**Published:** 2021-09-22

**Authors:** Adrian P Wiegmans, Ambber Ward, Ekaterina Ivanova, Pascal H G Duijf, Mark N Adams, Idris Mohd Najib, Romy Van Oosterhout, Martin C Sadowski, Greg Kelly, Scott W Morrical, Ken O’Byrne, Jason S Lee, Derek J Richard

**Affiliations:** Queensland University of Technology (QUT), Cancer and Ageing Research Program, Centre for Genomics and Personalised Health, School of Biomedical Sciences, Translational Research Institute, Woolloongabba QLD 4121, Australia; School of Medicine, University of Queensland, Herston, QLD, 4006, Australia; School of Medicine, University of Queensland, Herston, QLD, 4006, Australia; Epigenetics and Diseases Laboratory, QIMR Berghofer Medical Research Institute, Herston, QLD, 4006, Australia; Queensland University of Technology (QUT), Cancer and Ageing Research Program, Centre for Genomics and Personalised Health, School of Biomedical Sciences, Translational Research Institute, Woolloongabba QLD 4121, Australia; Queensland University of Technology (QUT), Cancer and Ageing Research Program, Centre for Genomics and Personalised Health, School of Biomedical Sciences, Translational Research Institute, Woolloongabba QLD 4121, Australia; Centre for Data Science, Queensland University of Technology (QUT), Brisbane, QLD, Australia; University of Queensland Diamantina Institute, University of Queensland, Brisbane, QLD, Australia; Queensland University of Technology (QUT), Cancer and Ageing Research Program, Centre for Genomics and Personalised Health, School of Biomedical Sciences, Translational Research Institute, Woolloongabba QLD 4121, Australia; Queensland University of Technology (QUT), Cancer and Ageing Research Program, Centre for Genomics and Personalised Health, School of Biomedical Sciences, Translational Research Institute, Woolloongabba QLD 4121, Australia; Epigenetics and Diseases Laboratory, QIMR Berghofer Medical Research Institute, Herston, QLD, 4006, Australia; Queensland University of Technology (QUT), Cancer and Ageing Research Program, Centre for Genomics and Personalised Health, School of Biomedical Sciences, Translational Research Institute, Woolloongabba QLD 4121, Australia; School of Medicine, University of Queensland, Herston, QLD, 4006, Australia; Epigenetics and Diseases Laboratory, QIMR Berghofer Medical Research Institute, Herston, QLD, 4006, Australia; Department of Biochemistry, Larner College of Medicine, University of Vermont, Burlington, VT 05405, USA; Queensland University of Technology (QUT), Cancer and Ageing Research Program, Centre for Genomics and Personalised Health, School of Biomedical Sciences, Translational Research Institute, Woolloongabba QLD 4121, Australia; School of Medicine, University of Queensland, Herston, QLD, 4006, Australia; Epigenetics and Diseases Laboratory, QIMR Berghofer Medical Research Institute, Herston, QLD, 4006, Australia; School of Biomedical Sciences, Queensland University of Technology, Brisbane, QLD, Australia; Queensland University of Technology (QUT), Cancer and Ageing Research Program, Centre for Genomics and Personalised Health, School of Biomedical Sciences, Translational Research Institute, Woolloongabba QLD 4121, Australia

The Authors wish to make the following corrections to their article (1).

Affiliations 2 and 3 have been corrected to:

^2^School of Medicine, University of Queensland, Herston, QLD, 4006, Australia

^3^Epigenetics and Diseases Laboratory, QIMR Berghofer Medical Research Institute, Herston, QLD, 4006, Australia

Jason S. Lee wishes to add another affiliation:

^7^School of Biomedical Sciences, Queensland University of Technology, Brisbane, QLD, Australia

The Publisher omitted the following joint authorship statement:

^‡^The authors wish it to be known that, in their opinion, the last two authors should be regarded as Joint Last Authors

The published article has been updated to reflect these corrections.
